# 16 years of topographic surveys of rip-channelled high-energy meso-macrotidal sandy beach

**DOI:** 10.1038/s41597-020-00750-5

**Published:** 2020-11-20

**Authors:** Bruno Castelle, Stéphane Bujan, Vincent Marieu, Sophie Ferreira

**Affiliations:** 1 CNRS/Univ. de Bordeaux, UMR EPOC, Talence, France; 2 CNRS/Univ. de Bordeaux, UMS POREA, Talence, France

**Keywords:** Geomorphology, Natural hazards, Physical oceanography

## Abstract

Sandy beaches are highly dynamic environments buffering shores from storm waves and providing outstanding recreational services. Long-term beach monitoring programs are critical to test and improve shoreline, beach morphodynamics and storm impact models. However, these programs are relatively rare and mostly restricted to microtidal alongshore-uniform beaches. The present 16-year dataset contains 326 digital elevation models and their over 1.635 × 10^6^ individual sand level measurements at the high-energy meso-macrotidal rip-channelled Truc Vert beach, southwest France. Monthly to bimonthly topographic surveys, which coverage progressively extended from 300 m to over 2000 m to describe the alongshore-variable changes, are completed by daily topographic surveys acquired during a 5-week field campaign. The dataset captures daily beach response at the scale of a storm to three large cycles of interannual variability, through the impact of the most energetic winter since at least 75 years and prominent seasonal erosion/recovery cycles. The data set is supplemented with high-frequency time series of offshore wave and astronomical tide data to facilitate its future use in beach research.

## Abstract (summary)

**Table:** 

Measurement(s)	Topography
Technology Type(s)	GPS navigation system
Factor Type(s)	temporal interval
Sample Characteristic - Environment	sandy beach
Sample Characteristic - Location	Aquitaine Region

Machine-accessible metadata file describing the reported data: 10.6084/m9.figshare.13070153

## Background & Summary

Sandy beaches buffer shores from storm waves and surge. They constantly evolve in response to storm, seasonal, and interannual variations in incident wave conditions^1,2^, anthropogenic forcings^3–5^, changes in natural sand supply^6,7^, to morphological adjustment of nearby tidal inlets or estuary mouth^8,9^, with sometimes a profound influence of geological settings^7,10–13^. Permanent video stations can infer daily shoreline proxy at reasonably low cost over years/decades^14,15^. However, the more costly sand level measurements can provide more accurate and richer insight into beach change through quantification of e.g. beach volume^16–18^, beach state and shape^19,20^ or shoreline position for different proxies in tidal environments^2,21^. Multi-annual/decadal high-resolution (months) topographic datasets of sandy beach systems are, however, scarce. They are limited to microtidal environments^22–24^, alongshore-uniform beaches^25–27^, and/or sectors influenced by coastal structures^28–30^ and/or by beach nourishment^30^. Monitoring programs of meso- to macro-tidal beaches are based on single of widely spaced transect(s)^17,31–33^, with the exception of the embayed beach of Perranporth, UK^34^. Depending on sand grain size and the tide range relative to modal wave conditions^35^, meso-macrotidal surf beaches can exhibit a strongly alongshore non-uniform intertidal and subaerial morphology due to the presence of rip channels, with a typical lengthscale of 100 s of meters^36^. Therefore, such beaches need to be surveyed with adequately spaced transects and large enough spatial coverage to comprehensively describe morphological changes.

**Fig. 1:**
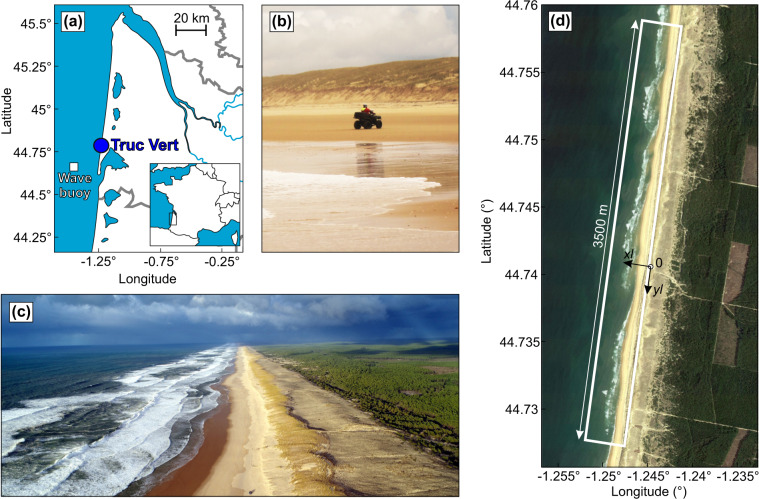
Survey site. (**a**) Location map of Truc Vert beach, southwest France with location of nearby wave buoy. (**b**) GNSS-equipped ATV used to perform the monthly to bimonthly surveys since 2003. (**c**) Aerial view showing the remote Truc Vert beach with prominent rip channels. (**d**) Survey zone and reference frame used at Truc Vert beach.

Truc Vert is a high-energy meso-macrotidal sandy beach located in southwest France (Fig. 1). It is a remote beach located more than three kilometers from the first inland carpark beach entry. Apart from the backing coastal dune which has been reprofiled in the early 80 s^37^, Truc Vert has never been nourished or affected by hard structures and tourism. This motivated the first topographic measurements in the late 90 s, surveying irregularly in time a single profile with a theodolite^38^. Only in 2003 when a RTK-GNSS receiver was acquired, equipping an ATV since 2005, did the surveys were set monthly and the spatial coverage increased to 300 m. The surveys subsequently became bimonthly, with the alongshore coverage increasing over the years before stabilizing at approximately 2200 m. This dataset was used, for instance, to develop beach state classification^39,40^, to address the intense beach-dune erosion and recovery from the outstanding winter of 2013/2014^17,18,41–43^ and to address the links between the dominant modes of climate variability on beach response^18^. It was also used to develop and improve a range of state-of-the art shoreline change models from the time scale of hours to decades^2,21,44,45^, to validate remotely-sensed shoreline estimation from publicly available satellite images^46^, to identify the morphological controls on spine injuries and drowning on surf beaches^47,48^, and to address the uncertainties in past and future multi-decadal shoreline evolutions^49,50^. In addition, a couple of intensive field experiments have been performed at Truc Vert beach^51^. In particular, in 2008 during the 5-week ECORS field measurements^52^ Truc Vert beach was surveyed daily to capture beach changes at the scale of a single storm^53^.

The bimonthly beach morphology survey program at Truc Vert exceeded 16 years in 2020. It covers a wide range of storms and of isolated extreme events^53^, an anomalous winter with severe storm clustering^17,41^ and a few cycles of large interannual beach variability. This monitoring program is also entering a new era as, since 2016, 4 km of coastal dunes are now surveyed at least quarterly^54,55^, together with notches dug in the dune to reinstate dune mobility and address plant community restoration along with biological monitoring^37^. With the growing interest in long-term beach datasets^23,30^ and growing need of validation data for a wealth of coastal models^56^, it is timely to facilitate the unrestricted use of this unique high-energy meso-macrotidal beach dataset, including the daily surveys during the ECORS’08 field campaign. The archived dataset includes all the raw survey points and suitably interpolated digital elevation models (DEMs), and time series of astronomical tide and offshore wave forcing. The beach survey dataset will be updated on a dedicated repository on an annual basis.

We anticipate that this dataset will be of interest for coastal modellers working on shoreline change, rip channel morphodynamics and beach erosion and recovery from the time scale of day to decades. In particular, with the recent development of tens of hybrid shoreline change models, this dataset is an ideal benchmark to test and compare shoreline models and perform ensemble-based simulations^56^ and probabilistic shoreline forecast. It will also be used for validation of satellite remote sensing data of coastal change, which is particularly challenging in meso-macrotidal energetic environments^46^.

## Methods

### Study site

The remote beach of Truc Vert is located in southwest France (Fig. 1a,c). The ocean wave exposed coastline of southwest France covers approximately 270 km of sandy beaches, which are backed by vegetated coastal dunes^57,58^, except along a few small coastal resorts^9^. The wave climate is generated in the north Atlantic Ocean predominantly by eastward tracking extra tropical cyclones^44,59^. Incident wave energy is strongly seasonally modulated with the monthly-averaged significant wave height *H*_*s*_ (peak wave period *T*_*p*_) ranging from 1.11 m (9 s) in July, with a dominant west-northwest direction, to 2.4 m (12.8 s) in January, with a dominant west direction^42^. Boreal winter wave activity and storms also show a strong interannual variability, with moderate winters alternating with extreme winters characterised by spatial and temporal severe storm clustering^17,41^. This large interannual variability is driven by natural modes of climate variability, particularly the West Europe Pressure Anomaly^60^. Positive WEPA reflects the intensification and southward shift of the sea level pressure gradient between the Azores high and the Icelandic low. This interannual variability in incident wave energy shows a significant increase in winter-mean wave height, variability, and periodicity over the last 70 years^61^.

Truc Vert beach is meso-macrotidal. The tide is semi-diurnal, with an annual mean spring tidal range of approximately 3.7 m and a largest astronomical tidal range of approximately 5 m^42^. Nearshore tide-driven currents are, however, negligible compared to wave-driven currents except for extremely low-energy waves and large tide range in the vicinity of the rip channels^62^.

The sediment consists primarily of medium quartz sand with a median diameter of approximately 350 *μ*m^63,64^. Beach sediment exhibits a large variability of 200–700 *μ*m correlated with a wide range of bedforms including bar/rip channels, cusps and megaripples^65^. Truc Vert beach is intermediate double barred^39^. The outer bar is subtidal and modally crescentic^66,67^, while the inner intertidal bar is classified modally as transverse bar and rip and tending to low tide terrace in summer^40^. The inner and outer bar mean rip spacing is approximately 400 and 700 m, respectively, although with large spatial and temporal variability^68^. The inner-bar rip channels enforce a large alongshore variability in beach morphology^43^ with megacusp embayment facing rip channels throughout the year. In contrast, the outer bar can drive larger scale beach variability under severe storms, which can potentially persist for years^41,43^.

Truc Vert beach is located in an approximately 20-km sector where the dune foot shoreline has been relatively stable over the last 70 years, in contrast with chronically eroding sectors further north^9^. However, the recent 2013/2014 winter, which was exceptional in terms of storminess and wave energy along the Atlantic coast of Europe^17,69^, caused severe beach and dune erosion at Truc Vert^41,42^. Erosion was highly variable alongshore, with the formation of localized megacusp embayments enforced by the outer bar variability in both depth and cross-shore position, with the erosion dune scarp height exceeding 6 m in its centre where the dune retreat reached 30 m.

### Topographic surveys

#### Monthly to bimonthly monitoring program

**Fig. 2:**
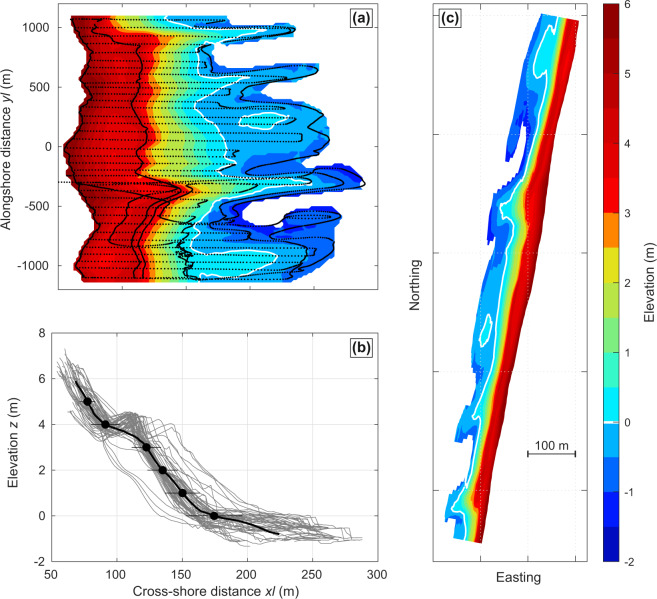
Example of topographic survey product. (**a**) Example of Truc Vert beach survey with colour bar and grey dots indicating AMSL elevation in metres and individual survey points, respectively, (**b**) superimposed 20-m spaced beach profiles (grey) and their alongshore average (black) with the horizontal error bars indicating the ±1 cross-shore standard deviation, (**c**) axis equal DEM.

Topographic surveys have been performed from September 2003 until December 2019, with a 1-year gap in 2008 due to equipment breakdown. This corresponds to 295 beach topographic surveys in 16.25 years. Topographic surveys were conducted at spring low tide using a RTK-GNSS by running cross-shore transects and, most of the time, a couple of alongshore transects depicting relevant features such as low tide mark, berm crest and dune toe (Fig. 2a). The distance between each transect varied roughly from 20 to 80 m, depending on local beach morphological patterns to be depicted and on the alongshore length covered. Most of the time, the inner bar was not entirely surveyed in the cross-shore direction as the water level was too high (wave runup) and/or the bar crest was too low. Sand level elevation was referenced to benchmarks of the French National Geodesic Service (NGF-IGN 69), which was transformed into elevation relative to MSL by substracting 0.4 m.

Spatial interpolation of sand level measurements is required to both generate a DEM on a given grid and to further compute the alongshore-averaged beach profile. For this, we first designed a regular grid in local coordinates (Fig. 1d), which has been used in most Truc Vert beach morphological studies^21,41–43,54,67^. In this local scheme, the cross-shore *xl* coordinate is positive onshore and the alongshore *yl* coordinate is positive to the south, with the xy origin being the ECORS’08 camera system position. The angle of rotation between the north and the *yl* axis in the local scheme is 10.8°. In order to accurately depict the prominent megacusps and rip channels at Truc Vert and to cope with occasional highly irregular survey transects (in both the cross-shore and alongshore directions, see for instance Fig. 2a), ordinary kriging with anisotropy^70^ was used. The semi-variogram model was based on the von Karman auto-covariance function, with an exponent *v* = 0.7. For each survey a mask was also designed which, together with anisotropic interpolation, prevented from the formation of spurious interpolation in the deep rip channels that were not surveyed, and spurious cuspate features with a spacing equalling that of the transects at the limits of the survey area. Interpolation was performed on a regular grid (Fig. 1d) with an alongshore and cross-shore mesh size of 20 m and 2 m, respectively, with an anisotropy ratio between *xl* and *yl* directions set to 10 (see an example of generated DEM in Fig. 2a,c). Such local scheme and interpolation technique enable to robustly address the alongshore variability in beach profile along the entire beach and to easily compute relevant alongshore-averaged beach proxies (Fig. 2b).

**Fig. 3:**
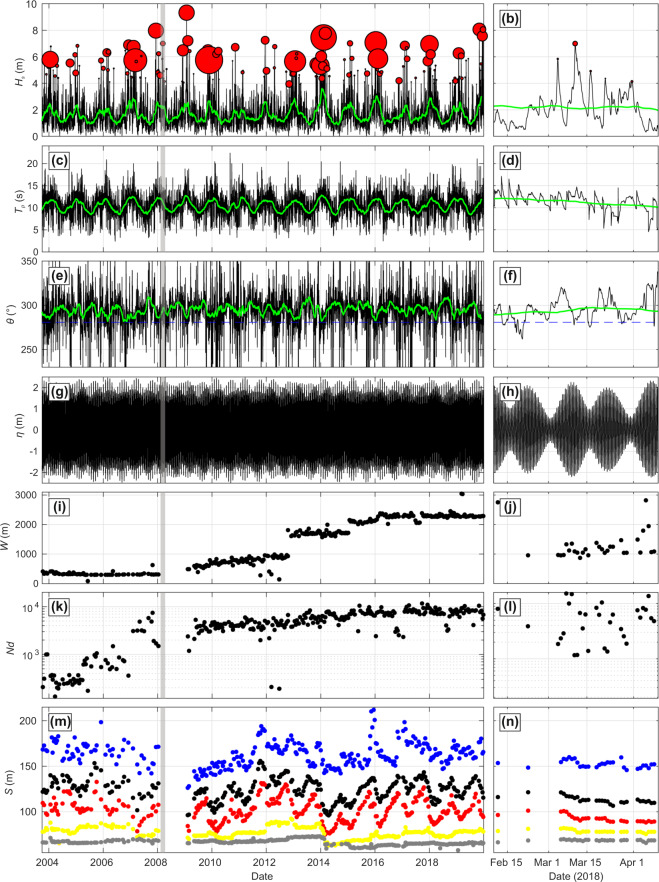
Time series products for the long-term monitoring program (left-hand panels) and ECORS’08 field experiment (right-hand panels). (**a**,**b**) *H*_*s*_ with bubbles indicating storm events (*H*_*s*_ > *H*_*s*95%_, whereby the size of the bubbles is proportional to storm duration based on *H*_*s*75%_ cut-off either size of storm maximum, (**c**,**d**) *T*_*p*_, (**e**,**f**) *θ* with their 2-month moving average shown in thick green) and in (**e**,**f**) the dashed blue line indicating shore-normal incidence at Truc Vert (280.8°). (**g**,**h**) (**b**) $\eta$, (**i**,**j**) survey alongshore coverage, (**k**,**l**) number of collected sand elevation points, (**m**,**n**) mean shoreline cross-shore position for different proxies *z* = 0, 2, 4 and 6 m.

The alongshore coverage increased over time in line with the evolution of the arising research questions (Fig. 3i). The alongshore coverage of the topographic surveys was about 350 m from 2003 to 2008, to between 600 and 1000 m from 2009 to 2013, to approximately 1700 m from 2013 to 2015, to approximately 2000 m in 2015 before stabilizing at approximately 2200 m since early 2016 (Fig. 3i). Since 2009, this corresponds to thousands of sand level measurements per survey, with an average of approximately 7500 since 2015 (Fig. 3k), for a total of over 1.456 × 10^6^ points. Figure 3m shows the time series of different shoreline proxies defined as the intersection of the alongshore-averaged profile with a given elevation datum. Seasonal cycles are prominent in the shoreline position for proxies *z* = 1.5 m and 3 m with typical amplitude of approximately 50 m. In contrast, shoreline proxy *z* = 6 m, which approximately corresponds to the dune toe position barely evolves and only retreated rapidly during the outstanding winter of 2013/2014.

#### ECORS’08 experiment

The ECORS’08 experiment was a 5-week large multi-institutional nearshore field experiment conducted at Truc Vert from March 3 to April 8, 2008^52^. During this experiment, almost daily topographic surveys were collected^71^. In addition to the ATV survey areas, a walking operator also sometimes collected topographic data in moderate water depths. Here we analysed the topographic dataset and the couple of topographic surveys performed just before this experiment using the same method as detailed above. The alongshore coverage of the surveys was irregular but was at least 1000 m (Fig. 3j), for a total of 31 surveys made on average of approximately 5800 individual sand elevation measurements (Fig. 3l). Due to the sequence of storms during the experiment, erosion of the upper part of the beach was observed, as well as large changes in alongshore variable morphology (not shown). This daily morphological dataset complements the monthly to bimonthly long term monitoring program.

### Waves

Because there is no continuous wave buoy measurements nearby Truc Vert covering the entire monitoring period, we resorted to 20 years of continuous hourly numerical wave hindcast (2000–2019) to estimate incident wave conditions. We used the grid point co-located with the CANDHIS directional wave buoy located (1° 26.8′W, 44° 39.15′N in Fig. 1a) moored in approximately 54-m depth, which intermittently collected directional wave measurements since 2001. Data from two spectral wave model Wavewatch III (WW3^72^) hindcasts implemented on the same unstructured grid were combined, with a resolution increasing from 10 km offshore to 200 m along the French Atlantic coast^73,74^. To further improve the accuracy of the wave forcing at Truc Vert, wave hindcast was corrected against the CANDHIS buoy measurements (see Section Technical Validation).

### Astronomical tides

Astronomical tides were derived at 10-min intervals using the tidal analysis package *T*_Tide_. A coastal hydrodynamic hindcast of the Bay of Biscay^75^ was used. Tide hindcast offshore of Truc Vert in approximately 7-m depth at spring low tide from January 2006 to January 2020 was used to perform the harmonic analysis. The tide time series was validated against 20 days of interspersed tide measurements offshore of Truc Vert during the ECORS’08 measurements.

## Data Records

The data can be obtained from the Open Science Framework Repository^76^. All files are in NetCDF format^77^, and provide detailed metadata for each variable within the file, using CF (Climate and Forecast) conventions 1.8 with Standard Name Table v75. The core data set in the repository is formed by 326 DEMs, all interpolated on the same regular grid, along with their raw sand level measurements from September 10, 2003 to December 26, 2019. Surveys performed in the frame of the long-running monitoring program are discriminated from those collected daily during the ECORS’08 experiment. The metadata of the topographic surveys are given in Table 1. This dataset is completed with hourly time series of wave conditions co-located with the offshore CANDHIS buoy in 54-m depth, and astronomical tide levels at 10-min interval (Table 2). Given that some shoreline change models need wave conditions up to 3 years prior to the start of shoreline measurements to accurately compute equilibrium conditions^2,21,50^, wave and tide time series start on January 1, 2000, that is, more than three years before the first topographic survey.

**Table 1:** Datasets.

Dataset	Parameter [unit]	Number of files	Data file(s)
Grid coordinates	long[°], latg[°], xlg[m], ylg[m], xg[m], yg[m]	1	Grids.nc
ECORS’08 topographic data	lon[°], lat[°], xl[m], yl[m], x[m], y[m], z[m], zg[m]	31	ECORS08_TrucVert_yyyy-mm-dd.nc
Monitoring topographic data	lon[°], lat[°], xl[m], yl[m], x[m], y[m], z[m], zg[m]	295	Monitoring_TrucVert_yyyy-mm-dd.nc

Beach surveys with individual elevation sand level measurements and DEMs.

**Table 2:** Datasets.

Dataset	Parameter [unit]	Time series	Sample frequency	Data file
Astronomical tide	tideTime[days], Tide[m]	01/2000–01/2020	10 min	Astronomical_Tide_TrucVert_2000–2020.nc
Waves	waveTime[days], waveHs[m], waveTp[s], waveDm[°]	01/2000–01/2020	1 hr	Waves_TrucVert_2000–2020.nc

Wave condition and astronomical tide.

## Technical Validation

### Topographic surveys

All sand level measurements were performed using a RTK-GNSS (Trimble 5700 then Trimble NetR9 for the reference receiver, and Trimble R6 then Trimble R8s for the mobile GNSS receiver) equipping the ATV (Fig. 1b). The horizontal and vertical accuracy is 8 mm and 15 mm, respectively. However, given that additional small errors can be due to e.g. antenna positioning, presence of ripples and megaripples, an accuracy of approximately 2.5 cm in the horizontal and 10 cm in the vertical is conservative. All surveys recorded the position of a fixed reference point on the top of dune, which was used for vertical and horizontal accuracy verification on each survey. All surveys were quality checked. Rare survey points outliers were manually removed. Eight topographic surveys were removed from the current dataset due to RTK-GNSS malfunctions and varying, irredeemable, elevation drift in time. The spatial interpolation to generate the DEM can also result in vertical errors. A comparison (not shown) on a 600-m stretch of Truc Vert beach of the DEM generated from a topographic survey performed on September 18, 2020 with that using high-resolution UAV-photogrammetry DEM^54^ performed the same day shows standard error of 7 cm. Therefore we estimate that the interpolation error is well under 10 cm. Raw sand level measurements are provided so that scientists can test other interpolation techniques.

### Wave hindcast

The HOMERE^73^ (1994–2017) and NORGAS-UG^74^ (2008–2019) hindcasts were combined. HOMERE and NORGAS-UG hindcasts have been extensively validated with directional buoys and satellite altimeters, showing excellent skill^73,74^. We provided additional correction by calibrating wave conditions offshore of Truc Vert against the wave buoy measurements. We used *in situ* wave data collected intermittently between January 2008 and October 2019 resulting in a total of approximately 8.5 years of hourly wave data (*n* = 73,672). Peak wave period, significant wave height and mean wave direction were linearly regressed against measurements. After correction, *H*_*s*_ hindcast shows strong agreement with measured wave data with for HOMERE (NORGAS-UG) a coefficient of determination *R*^2^ = 0.94 (0.93) and a root mean square error RMSE = 0.25 m (0.26 m). Accuracy decreases for *T*_*p*_ (*R*^2^ = 0.25, RMSE = 1.7 s for HOMERE and *R*^2^ = 0.37, RMSE = 1.6 s for NORGAS-UG). The decrease in accuracy for *T*_*p*_ can be partly explained by discontinuities in measured and modelled peak wave period data, which can vary substantially under mixed sea/swell regimes and when there is bimodality in the wave spectra. Corrected mean wave direction hindcast showed good agreement, with *R*^2^ = 0.71, RMSE = 9.5° for both HOMERE and NORGAS-UG. The resulting continuous corrected wave time series combines HOMERE (2000–2017) and NORGAS-UG (2018–2019) corrected hindcasts.

### Tide levels

Tide hindcast was validated against approximately 20 days of interspersed water level measurement at 5-min interval collected at Truc Vert during the ECORS’08 measurements. Given that these measurements also include non-tidal residuals, the non-tidal residuals of the hindcast were included in the comparison. Water levels show a very good agreement with measured tide with *R*^2^ = 0.99 and RMSE = 0.12 m. It is important to note that this validation was characterised by high waves and storm winds, with storm surge reaching 0.22 m.

## Usage Notes

### Tide data

The astronomical tide data does not take into account non-tidal effects on sea levels such as storm surge, which can be estimated using coastal model hindcast. The 2006–2020 hindcast from the MARC platform indicates that such non tidal effects in water level at Truc Vert are small, with a standard deviation of 0.09 m and a maximum of 0.6 m occurring on February 28, 2010 at 2AM during the peak of storm Xynthia. In addition, in order to estimate shoreline water levels at the shore, the user must account for wave setup or runup that is not observed in such water level hindcast, but can be estimated from the wave time series with wave set-up or runup empirical formulas^78^.

### Wave data

Wave conditions are provided approximately 10 km from the coast in 54-m depth, offshore of Truc Vert. Given that the offshore bathymetry is essentially uniform alongshore, wave dissipation and alongshore variability of inshore wave energy enforced by offshore wave refraction is limited. Therefore wave conditions computed at the Candhis buoy can be considered as a good proxy for the wave conditions arriving at Truc Vert^42^. Estimation of breaking wave conditions can however be performed using computationally cheap empirical formula (e.g^79^) owing to the shore-parallel bathymetric iso-contours, like for instance in^50^.

### Sand elevation data

The DEMs provide ready-to-use binned morphological data on a relevant local grid, which can be easily processed to derive a wealth of alongshore-variable or alongshore-averaged morphological proxies. However, the raw sand elevation data provided along with the DEMs can be used directly or combined with other interpolation techniques.

### Complementary dataset

Additional wave buoy data at http://candhis.cetmef.developpement-durable.gouv.fr/. Additional tide data at http://refmar.shom.fr/en/ARCACHON_EYRAC. Additional sea level and storm surge hindcast from the MARC platform (Modelling and Analysis for Research in Coastal environment) at https://marc.ifremer.fr. Additional Lidar topographic data at http://www.observatoire-cote-aquitaine.fr/Mise-en-ligne-de-l-ensemble-des-millesimes-LiDAR. Bathymetric data collected during the ECORS’08 field experiment at Truc Vert can be provided by the first author upon request.

## Data Availability

All data files created and used in processing are formatted in the Network Common Data Form (NetCDF), providing detailed metadata for each variable within the file, and can be read using MATLAB, Python, Fortran, C, C++, Java, and other languages. A Code file used to interpolate the raw sand elevation data is included in the repository folder. Code is written in MATLAB (R2019a) and is fully commented. Although MATLAB is a proprietary language, the.m files can be read with a text viewer.
